# Why dementia is often not diagnosed by subtype: care pathways and imaging access in the United Arab Emirates

**DOI:** 10.3389/fpsyt.2026.1910567

**Published:** 2026-08-05

**Authors:** Syed Fahad Javaid, Fadwa Al Mugaddam, Zubaida Shebani, Syed Ali Bokhari

**Affiliations:** 1 Department of Psychiatry, College of Medicine and Health Sciences, United Arab Emirates University, Al Ain, United Arab Emirates; 2 Department of Behavioral Medicine, College of Medicine and Health Sciences, Sultan Qaboos University, Muscat, Oman; 3 Al Amal Psychiatric Hospital, Emirates Health Services, Dubai, United Arab Emirates

**Keywords:** behavioural and psychological symptoms of dementia, care pathways, dementia, diagnostic completeness, mental health services research, neuroimaging, old age psychiatry, United Arab Emirates

## Abstract

**Background:**

Whether a person with dementia receives a specific subtype diagnosis rather than a nonspecific label is a marker of diagnostic completeness. Subtype shapes safe management, including psychotropic prescribing, behavioural and psychological symptom management, and eligibility for Alzheimer’s disease therapies, yet nonspecific labels remain common where specialist assessment is unevenly accessed. We examined which patient, clinical, and service-pathway factors were associated with specific subtype assignment in a dementia cohort in the United Arab Emirates (UAE), and whether an exploratory model could help identify patients who might remain without a subtype. The outcome reflects the specificity of the recorded diagnosis, not adjudicated accuracy.

**Methods:**

We conducted a retrospective cohort study of patients with dementia seen at the Behavioural Sciences Institute, Al Ain Hospital from 1 January 2010 to 31 December 2019. The primary outcome was specific subtype assignment versus unspecified dementia. Factors examined were age, sex, nationality, brain imaging utilisation, comorbidity burden, symptom cluster, location of first assessment, and year. We fitted multivariable logistic regression with internal validation, sensitivity analyses, multiple imputation, scanned-subgroup analysis, and decision-curve analysis.

**Results:**

Of 825 patients, 786 entered the complete-case analytic sample; 336 (42.7%) had an unspecified diagnosis and 450 (57.3%) received a specific subtype label. Brain imaging showed the strongest adjusted association with specific subtype assignment (adjusted odds ratio = 4.00; 95% confidence interval = 2.84–5.63). Lower odds of specific assignment were associated with three or more comorbidities (0.40, 0.29–0.57), behavioural presentation (0.60, 0.39–0.94), emergency department route (0.29, 0.16–0.51), other route (0.31, 0.20–0.48), and neurology inpatient route (0.44, 0.22–0.90). Among scanned patients, multimorbidity and nonclinic routes remained associated with unspecified labelling. The prescan model showed positive net benefit across thresholds of 0.20–0.50.

**Conclusions:**

Diagnostic completeness was associated less with demographics than with imaging access, clinical complexity, and route of entry to care. Maintaining access to imaging within dementia pathways, and to timely specialist psychiatric assessment for acute and nonspecialist routes, may be associated with more complete subtype recording in the UAE and comparable Gulf systems. An exploratory prescan model identified patients at risk of remaining without a recorded subtype, though external validation is required before clinical use.

## 1 Introduction

Dementia is a growing priority for health systems worldwide, and the number of people affected is projected to rise steeply over the coming decades (1). Improving dementia awareness and developing dementia-friendly health systems are also recognised public-health priorities (2). Dementia is also a central concern of older-adult mental-health services (3): behavioural and psychological symptoms affect most individuals with dementia during the course of their illness, and psychiatric services play a critical role in their assessment and management (4).

As provision expands, the quality of dementia care depends not only on whether dementia is detected but also on how completely it is characterised. Dementia is not a single clinical entity: Alzheimer’s disease is the most common cause, but vascular dementia, dementia with Lewy bodies, frontotemporal dementia, Parkinson’s disease dementia, mixed dementia, and less common syndromes differ in natural history, symptom profile, prognosis, and management priorities. Moving beyond a broad dementia label towards specific subtype assignment is therefore clinically important for counselling, vascular risk management, symptomatic treatment, follow-up planning, and access to subtype-specific or disease-modifying therapies. It also has direct consequences for psychiatric prescribing and patient safety: individuals with dementia with Lewy bodies are at risk of severe antipsychotic sensitivity reactions, so an unrecognised subtype can lead to avoidable harm when behavioural symptoms are treated (5). Specific subtype assignment depends on a care pathway that includes the right investigations, specialist interpretation, and documentation; it can therefore be read as a marker of diagnostic completeness. For psychiatry, this is not only a documentation issue but also a determinant of safe and effective care, because subtype guides psychotropic prescribing, shapes the interpretation and management of behavioural and psychological symptoms, and informs counselling and prognosis. Identifying what is associated with diagnostic completeness is therefore a mental-health services question with direct implications for how dementia care is organised, resourced, and delivered.

Contemporary guidance treats structural brain imaging as integral to dementia assessment when subtype is not already clear: the National Institute for Health and Care Excellence (6), the American Academy of Neurology (7), and the American College of Radiology (8) all recommend computed tomography or magnetic resonance imaging in the initial work-up. Beyond excluding structural mimics, neuroimaging supports differentiation by identifying patterns of atrophy, vascular burden, and regional involvement (9).

Prior studies show that nonspecific dementia coding is more frequent where specialist access is limited (10, 11) and where dementia is recorded in general hospital settings (12, 13), and a recent systematic review on dementia misdiagnosis emphasised that delayed or incorrect subtype recognition can restrict access to appropriate care (14). Subtype assignment should therefore be understood as an outcome of service organisation, investigation access, and coding practice.

Dementia epidemiology in Arab countries remains comparatively underdescribed (15), whilst recent work has highlighted substantial economic burden and persistent service gaps (16). In the United Arab Emirates (UAE), emerging evidence suggests diagnosis may be delayed by several years after symptom onset (17); however, to our knowledge, no peer-reviewed study from the UAE or the wider Gulf has examined the completeness of dementia subtype recording or the service-pathway factors that shape it, leaving a gap of both regional and international relevance. Al Ain Hospital serves a large catchment population, making this cohort an informative window into real-world diagnostic pathways in the UAE and the wider Gulf region.

The present study examined determinants of specific dementia subtype assignment versus an unspecified label in a retrospective hospital cohort from the Behavioural Sciences Institute of Al Ain Hospital between 2010 and 2019. The primary aim was to identify the patient, clinical, investigation-related, and service-pathway factors associated with receiving a specific subtype label, with particular attention to modifiable health-system factors such as imaging access and route of first assessment. Secondary aims were to characterise model performance and internal validity, test robustness across sensitivity and imputation analyses, and evaluate whether an exploratory prescan model could help identify patients who might benefit from additional diagnostic work-up, using decision-curve analysis. Throughout, subtype assignment is interpreted as a marker of diagnostic completeness and documentation within the mental-health and dementia care pathway, rather than as a measure of underlying neuropathological accuracy or of the diagnostic decision-making of individual clinicians. We frame the question as one of mental-health services research, given its direct bearing on psychiatric management, prescribing safety, and the quality of care for older people with dementia.

## 2 Materials and methods

### 2.1 Study design and setting

This retrospective cohort study analysed clinical records of patients with a documented dementia diagnosis seen at the Behavioural Sciences Institute (BSI) of Al Ain Hospital over the period from 1 January 2010 to 31 December 2019. The recorded date of dementia diagnosis, from which the year covariate and the annual-proportion analysis were derived, ranged from 2008 to 2021 for a small number of records; the annual-proportion analysis was therefore restricted to 2010–2019 (*n* = 767), the years with adequate case numbers, whereas all regression models used the full complete-case sample (*n* = 786). Al Ain had a 2024 population estimate of 986,910, with the hospital serving a large catchment population across Al Ain City and surrounding areas (18).

### 2.2 Participants

**Figure 1:**
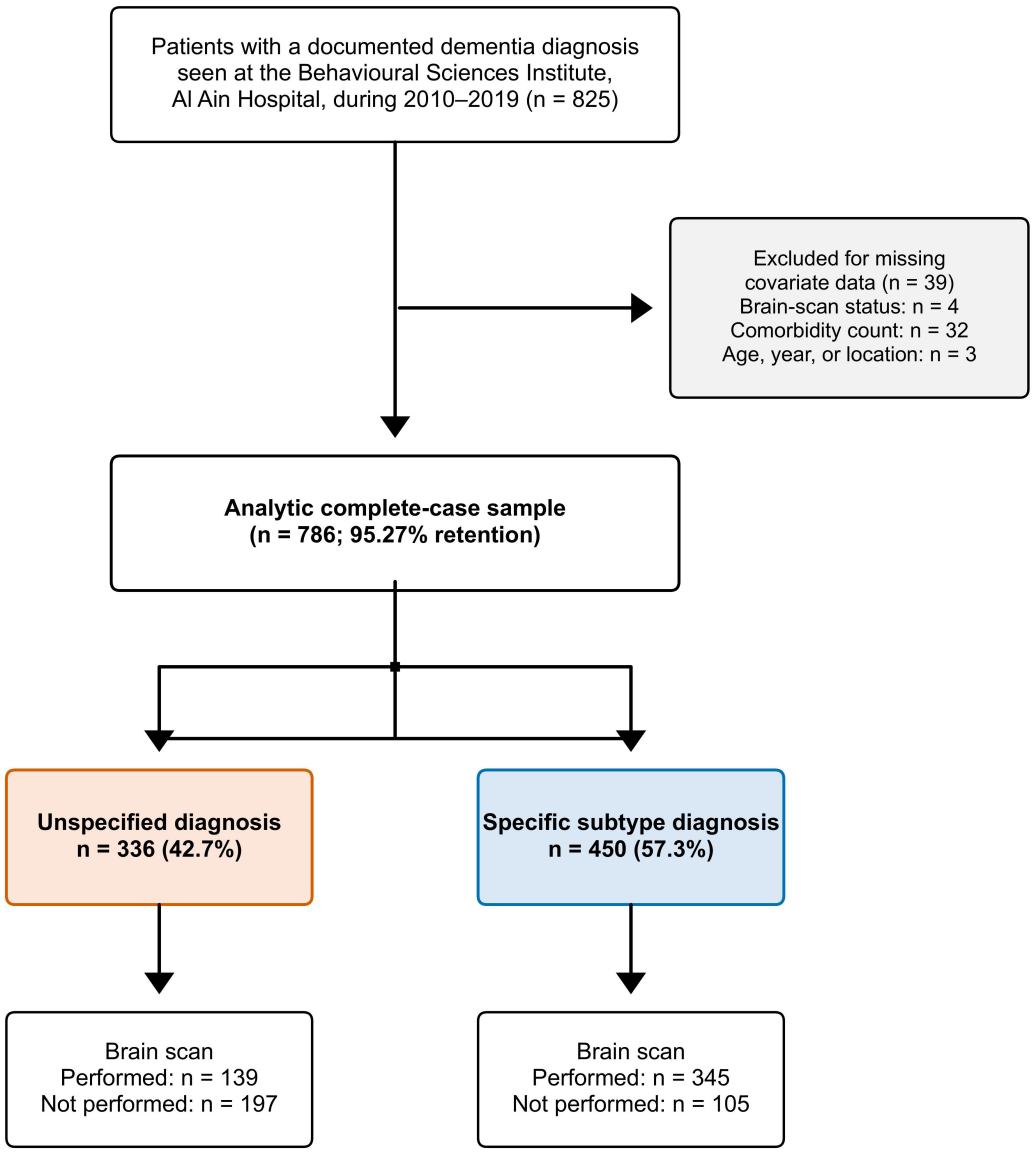
Participant flow and subtype-determination status, 2010–2019. From 825 patients meeting the inclusion criteria, 39 were excluded due to missing covariate data, yielding an analytic complete-case sample of *n* = 786 (95.27%). Structural brain imaging utilisation within each diagnostic outcome is presented in the lower row.

Eligible cases were patients aged 18 years or older with a documented dementia diagnosis at BSI within the window. Patients with a primary diagnosis of intellectual disability, traumatic brain injury, acute cerebrovascular accident as the principal admission diagnosis, or any concurrent mental disorder as the principal diagnosis were excluded; vascular dementia was retained as a specific subtype. Of 825 patients meeting inclusion criteria, 39 were excluded for missing covariate data, yielding a complete-case sample of 786 patients (95.27% retention; **Figure 1**).

### 2.3 Outcome

The primary outcome was specific dementia subtype assignment, recoded as binary (specific subtype = 1; unspecified = 0). Specific subtypes comprised Alzheimer’s disease, vascular, Parkinson’s disease, Lewy body, frontotemporal, mixed, and posterior cortical atrophy dementias. All subtype labels were clinician-entered diagnostic terms extracted unchanged from the electronic clinical record. Diagnoses were recorded as free-text clinical labels rather than as coded entries, so no classification version or code mapping applies; the outcome reflects the specificity of the recorded diagnosis, not adjudicated neuropathological accuracy. Categorisation was therefore phenotypic and syndromic, reflecting the diagnostic labels applied in routine care in a setting without routine molecular or imaging biomarker confirmation. Frontotemporal dementia was recorded as a single category without a behavioural- or language-variant distinction, and primary progressive aphasia did not appear as a recorded label in this cohort and was therefore neither assigned nor actively excluded.

The full coding scheme for outcome and covariates is provided in **Supplementary Table 1**. Six records had a missing dementia-subtype label; consistent with the parent cohort definition, these were treated as unspecified dementia, an assumption examined in a strict sensitivity analysis that excluded them. Four of the six were present in the complete-case sample (the other two were excluded for missing covariates), so the strict analysis comprised 782 patients. Duration of undiagnosed symptoms was retained as a descriptive variable in **Table 1** but was not modelled as a covariate or outcome.

**Table 1:** Sample characteristics by subtype determination (analytic sample, *n* = 786).

Variable	Unspecified (*n* = 336)	Specific (*n* = 450)	Effect size		*p*-value
			*d*	*V*	
Age at diagnosis (years)	79.4 ± 10.8 [median = 80.0; IQR = 72.0–86.0]	77.4 ± 11.0 [median = 77.0; IQR = 70.0–83.0]	0.18		0.001^*^
Duration of undiagnosed symptoms (months)	34.4 ± 29.4 [median = 24.0; IQR = 12.0–48.0]	34.8 ± 28.1 [median = 24.0; IQR = 12.0–48.0]	− 0.01		0.526
Year of diagnosis	2015 ± 3 [median = 2016; IQR = 2013–2018]	2015 ± 3 [median = 2015; IQR = 2013–2017]	0.08		0.130
Female sex (*n*; %)	181 (53.9)	234 (52.0)		0.02	0.655
Emirati nationality (*n*; %)	226 (67.3)	268 (59.6)		0.08	0.033^*^
Brain scan performed (*n*; %)	139 (41.4)	345 (76.7)		0.36	< 0.001^*^
≥ 3 comorbidities (*n*; %)	218 (64.9)	202 (44.9)		0.20	< 0.001^*^
Age ≥ 65 years (*n*; %)	316 (94.0)	404 (89.8)		0.07	0.045^*^
Initial symptom cluster (*n*; %)					
Affective	9 (2.7)	38 (8.4)		0.16^*^	< 0.001^*^
Behavioural	79 (23.5)	66 (14.7)			
Memory-led	195 (58.0)	276 (61.3)			
Psychotic	17 (5.1)	26 (5.8)			
Vegetative/motor	36 (10.7)	44 (9.8)			
Location of first assessment (*n*; %)					
CL	7 (2.1)	23 (5.1)		0.32^*^	< 0.001^*^
ED	50 (14.9)	43 (9.6)			
Neurology clinic	39 (11.6)	86 (19.1)			
Neurology inpatient	19 (5.7)	30 (6.7)			
Other	173 (51.5)	115 (25.6)			
Psychiatry clinic	47 (14.0)	148 (32.9)			
Psychiatry inpatient	1 (0.3)	5 (1.1)			

Continuous variables are reported as mean ± SD, with median and interquartile range in square brackets; between-group comparison was performed using the Mann–Whitney *U* test. Categorical variables are reported as *n* (%); comparison was performed using Pearson’s Chi-square or Fisher’s exact test, where appropriate. Effect sizes: Cohen’s *d* for continuous variables (*d*); Cramer’s *V* for categorical variables (*V*). Age ≥ 65 years is reported descriptively and was not entered into the primary multivariable model, which used age at diagnosis as a continuous covariate.

*DUS*, duration of undiagnosed symptoms.

^*^ *p* < 0.05**—**statistical significance.

### 2.4 Covariates

Covariates were specified in advance and comprised age at diagnosis (continuous), sex, nationality (Emirati versus non-Emirati), structural brain imaging utilisation (yes versus no; a completed structural scan documented in the record; imaging modality, computed tomography versus magnetic resonance imaging, was not systematically recorded in the source data and therefore could not be analysed, and imaging was therefore operationalised as a binary indicator), comorbidity load (three or more versus one to two conditions, as collapsed in the source extract, so no underlying count was available; no record carried a zero category, and the 32 records with no condition listed had a blank category and were excluded as missing, giving a minimum of one condition in the analytic sample; the recorded conditions were diabetes mellitus, hypertension, cerebrovascular disease, falls or mobility problems, chronic kidney disease, parkinsonism, chronic liver disease, a recorded mental-health condition, and other), initial symptom cluster (memory-led, behavioural, psychotic, affective, vegetative or motor; reference: memory-led), location of first assessment (psychiatry clinic, psychiatry inpatient, neurology clinic, neurology inpatient, emergency department, consultation-liaison, other; reference: psychiatry clinic), and year of diagnosis (continuous, centred on 2015). Initial presenting symptoms were recorded using a study-specific scheme applying 11 Neuropsychiatric Inventory Questionnaire (NPI-Q) domain labels together with an additional memory category (forgetfulness) that is not an NPI-Q domain; the NPI-Q elation/euphoria domain was not recorded in any case. Symptoms were grouped into five clusters: memory-led (forgetfulness); behavioural (agitation/aggression, irritability/lability, disinhibition); psychotic (delusions, hallucinations); affective (depression/dysphoria, anxiety, apathy/indifference); and vegetative/motor (motor disturbance, appetite/eating, nighttime behaviours) (19). The full mapping is given in **Supplementary Table 1**.

### 2.5 Statistical analysis

Descriptive comparisons used Mann–Whitney *U* tests for continuous variables and chi-square or Fisher’s exact tests for categorical variables, with Cohen’s *d* and Cramer’s *V* as effect sizes alongside two-sided *p*-values.

Univariate logistic regression was conducted for each candidate covariate, with unadjusted odds ratios for all covariates presented in **Supplementary Figure 1**; a primary multivariable model then entered all covariates simultaneously to return adjusted odds ratios (aORs) with 95% confidence intervals (CIs). All odds ratios are expressed for the outcome of specific subtype assignment, so that an aOR above 1 indicates higher odds of a specific subtype and an aOR below 1 indicates lower odds, equivalently higher odds of an unspecified label given by the reciprocal. Multicollinearity was assessed by variance inflation factors (target threshold of 5). Calibration was assessed by the Hosmer–Lemeshow test and a decile calibration plot; discrimination by the area under the curve (AUC) with nonparametric bootstrap 95% CI based on 2,000 attempted resamples. Apparent and optimism-corrected AUC values used Harrell’s bootstrap (**Supplementary Table 2**). A 10-fold cross-validated L1-penalised (LASSO) logistic regression provided a supportive assessment of covariate stability (**Supplementary Table 3**); model diagnostics are summarised in **Supplementary Table 4**.

A prespecified analysis restricted to patients with documented brain imaging fitted the same logistic specification with brain scan held constant, characterising factors associated with subtype assignment amongst patients with documented brain imaging. A prescan logistic model excluding brain scan was also fitted to enable decision-curve analysis (20) after reverse-coding the outcome so that higher predicted probabilities represented risk of an unspecified diagnosis. Net clinical benefit was evaluated against work-up all and work-up none reference strategies; additional subtype-determination work-up was operationally defined as completion of structural imaging where not yet performed, memory clinic review, postacute structured diagnostic review, coding review, or multidisciplinary team review. Decision-curve analysis does not require monetary cost inputs; instead, the threshold probability represents the harm-to-benefit trade-off, that is, the probability of an unspecified diagnosis at which a service would be indifferent between undertaking and withholding additional work-up. A threshold of 0.40, for example, corresponds to accepting up to 1.5 unnecessary work-ups for each patient correctly identified as likely to remain unspecified.

Robustness analyses were specified before model fitting. As the annual proportion of unspecified diagnoses was not monotonic across the study window, the temporal pattern was assessed with a likelihood-ratio test of between-year heterogeneity (calendar year entered as a categorical term) and a logistic model with linear and quadratic year terms to characterise curvature, rather than a test assuming a monotonic trend. Year-by-brain-scan and symptom-cluster-by-brain-scan interactions were examined via likelihood-ratio tests (**Supplementary Figures 2**, **S3**). Two alternative outcome definitions, strict missing-data handling, and exclusion of patients aged over 100 years were examined as sensitivity scenarios (**Supplementary Table 5**). Comorbidity-count missingness was addressed by multiple imputation using iterative chained equations (m = 20), with pooled estimates obtained under Rubin’s rules (21, 22) (**Supplementary Table 6**). An exploratory product-of-coefficients analysis examined whether the behavioural-versus-memory-led association was partly expressed through brain-scan ordering, without assuming a formal counterfactual decomposition (**Supplementary Figure 4**). The prescan model specification is reported in **Supplementary Table 7**. The fitted pre-scan logistic model used for the decision-curve analysis took the form:


$$\text{logit}\;(P(\text{unspecified}=1))=\;-2.011\;+\;0.001\cdot \text{Age}\;+\;0.207\cdot \text{Female }+\;0.350\cdot \text{Emirati }+\;0.795\cdot {\text{Comorb}}_{3+}\;+\;0.587\cdot \text{Behavioural }+\;0.025\cdot \text{Psychotic }-\;0.600\cdot \text{Affective }+\;0.158\cdot \text{Vegetative}/\text{motor }-\;0.623\cdot \text{Psych}\_\text{inpatient }+\;0.263\cdot \text{Neuro}\_\text{clinic }+\;0.640\cdot \text{Neuro}\_\text{inpatient }+\;1.377\cdot ED\;+\;1.450\cdot \text{Other }-\;0.177\cdot \text{CL}\;-\;0.007\cdot \left(\text{Year}-2015\right)$$

Analyses were conducted in Python 3.13 using statsmodels 0.14.4, scikit-learn 1.6.1, scipy 1.15.3, and matplotlib 3.10.0. Reporting follows the STROBE guideline for cohort studies (23), with TRIPOD elements for the prediction-model components (24). Two-sided *p*-values below 0.05 were considered statistically significant.

### 2.6 Ethical considerations

Ethical approval was granted by the Al Ain Hospital Research Ethics Governance Committee (Approval No. AAHEC-11-20-030). As this was a retrospective analysis of routinely collected clinical records, the requirement for individual informed consent was waived. All data were extracted and analysed in de-identified form. The study was conducted in accordance with the Declaration of Helsinki and relevant institutional requirements for the use of clinical data in health research.

## 3 Results

### 3.1 Sample characteristics

Of the 825 patients with a documented dementia diagnosis seen at the Behavioural Sciences Institute between 2010 and 2019, 786 contributed complete covariate data for the primary model (**Figure 1**). Exclusions were due to missing comorbidity count (*n* = 32), missing brain-scan status (*n* = 4), and missing age, year of diagnosis, or location (*n* = 3). Within the analytic sample, 336 patients (42.7%) had an unspecified diagnosis, and 450 (57.3%) received a specific subtype label. Sample characteristics by subtype-determination status are presented in **Table 1**.

Patients in the unspecified group were marginally older at diagnosis (mean = 79.4 years; SD = 10.8) than those receiving a specific subtype (mean = 77.4 years; SD = 11.0; Mann–Whitney *p* = 0.001; Cohen’s *d* = 0.18). Female sex (53.9% versus 52.0%) did not differ between groups (*p* = 0.655). Emirati nationality was modestly more frequent in the unspecified group (67.3% versus 59.6%; *p* = 0.033; *V* = 0.08). Duration of undiagnosed symptoms did not differ between groups (median = 24 months in both; IQR = 12 to 48; *p* = 0.526), and no association was detected between subtype determination and the timing of clinical recognition in this cohort.

Three factors showed substantially larger between-group differences. Brain imaging utilisation was 41.4% in the unspecified group versus 76.7% in the subtype-determined group (*V* = 0.36; *p* < 0.001). Three or more comorbidities were present in 64.9% of the unspecified group versus 44.9% of the subtype-determined patients (*V* = 0.20, *p* < 0.001). The most frequently recorded conditions were hypertension, diabetes mellitus, falls or mobility problems, and cerebrovascular disease, and comorbidity burden was higher in the unspecified group (mean = 2.53 versus 2.24 recorded conditions; *p* < 0.001). Therefore, it was not balanced between groups and was retained as a covariate (**Supplementary Table 8**). The distribution of locations of first assessment also differed (*V* = 0.32; *p* < 0.001), with the unspecified group disproportionately presenting through the other (51.5% versus 25.6%) and emergency department (14.9% versus 9.6%) routes, whilst the subtype-determined patients were overrepresented at the psychiatry clinic (32.9% versus 14.0%) and the neurology clinic (19.1% versus 11.6%). Behavioural presentations were more common in the unspecified group (23.5% versus 14.7%), whereas affective presentations were more common in the subtype-determined group (8.4% versus 2.7%).

### 3.2 Factors associated with subtype determination

Univariate and adjusted logistic regression results are presented in **Table 2**. After mutual adjustment, statistically significant and clinically substantial adjusted associations were present across four covariate domains: brain imaging, comorbidity burden, behavioural presentation, and location of first assessment. Brain imaging utilisation was the strongest correlate (aOR = 4.00; 95% CI = 2.84, 5.63; *p* < 0.001), corresponding to approximately fourfold higher odds of subtype determination amongst scanned patients. A comorbidity load of three or more conditions was inversely associated with subtype determination (aOR = 0.40; 95% CI = 0.29, 0.57; *p* < 0.001), and behavioural presentations were associated with reduced odds of subtype determination relative to memory-led presentations (aOR = 0.60; 95% CI = 0.39, 0.94; *p* = 0.025). The location of first assessment had independent effects, with three categories showing reduced odds relative to the psychiatry clinic. Patients first assessed through the emergency department had the lowest adjusted odds (aOR = 0.29; 95% CI = 0.16, 0.51; *p* < 0.001; equivalent to a 3.50-fold higher odds of an unspecified label), followed by those first assessed through the other category(aOR = 0.31; 95% CI = 0.20, 0.48; *p* < 0.001) and the neurology inpatient setting (aOR = 0.44; 95% CI = 0.22, 0.90; *p* = 0.024). Age, sex, Emirati nationality, year of diagnosis, and the psychotic, affective, and vegetative-motor symptom clusters did not retain independent effects.

**Table 2:** Univariate and adjusted logistic regression analyses for factors associated with specific subtype determination.

Variable	Unadjusted OR	Unadjusted 95% CI	Unadjusted *p*	Adjusted OR	Adjusted 95% CI	Adjusted *p*
Age at diagnosis (per year)	0.983	[0.971, 0.996]	0.012	1.01	[0.99, 1.02]	0.496
Brain scan performed	4.66	[3.42, 6.34]	< 0.001	4.00	[2.84, 5.63]	< 0.001
≥ 3 comorbidities	0.44	[0.33, 0.59]	< 0.001	0.40	[0.29, 0.57]	< 0.001
Emirati nationality	0.72	[0.53, 0.96]	0.027	0.76	[0.54, 1.07]	0.114
Female sex	0.93	[0.70, 1.23]	0.604	0.85	[0.61, 1.17]	0.315
Location of first assessment: CL	1.04	[0.42, 2.59]	0.927	1.13	[0.42, 3.05]	0.807
Location of first assessment: ED	0.27	[0.16, 0.46]	< 0.001	0.29	[0.16, 0.51]	< 0.001
Location of first assessment: neurology clinic	0.70	[0.42, 1.16]	0.163	0.81	[0.47, 1.41]	0.459
Location of first assessment: neurology inpatient	0.50	[0.26, 0.97]	0.041	0.44	[0.22, 0.90]	0.024
Location of first assessment: other	0.21	[0.14, 0.32]	< 0.001	0.31	[0.20, 0.48]	< 0.001
Location of first assessment: psychiatry inpatient	1.59	[0.18, 13.93]	0.677	1.65	[0.16, 16.65]	0.671
Symptom cluster: affective	2.98	[1.41, 6.31]	0.004	1.60	[0.70, 3.68]	0.266
Symptom cluster: behavioural	0.59	[0.41, 0.86]	0.006	0.60	[0.39, 0.94]	0.025
Symptom cluster: psychotic	1.08	[0.57, 2.05]	0.812	0.89	[0.43, 1.84]	0.751
Symptom cluster: vegetative/motor	0.86	[0.54, 1.39]	0.547	0.76	[0.44, 1.32]	0.336
Year of diagnosis (per year, centred)	0.97	[0.92, 1.02]	0.294	0.99	[0.93, 1.06]	0.829

Outcome: assignment of a specific dementia subtype (unspecified as the reference). The univariate column reports unadjusted odds ratios from single-covariate models; the adjusted column reports estimates from the primary multivariable model that entered all covariates simultaneously. Reference categories: female sex (male), Emirati (non-Emirati), brain scan (no), three or more comorbidities (one to two), symptom cluster (memory-led), location (psychiatry clinic). Year of diagnosis is centred on the sample median (2015). All *p*-values are two-sided. The unadjusted age odds ratio and confidence interval are reported to three decimal places because rounding to two decimal places does not resolve a confidence limit that lies close to, but excludes, 1. For covariates with an adjusted odds ratio below 1, the reciprocal expresses the odds of an unspecified label: three or more comorbidities: 2.48 (95% CI = 1.76, 3.47); behavioural cluster: 1.66 (95% CI = 1.07, 2.58); emergency department: 3.50 (95% CI = 1.96, 6.25); other route: 3.25 (95% CI = 2.08, 5.10); Neurology inpatient: 2.25 (95% CI = 1.11, 4.56). Reciprocals are derived from unrounded model estimates, with confidence limits inverted and transposed.

### 3.3 Model performance and internal validity

**Figure 2:**
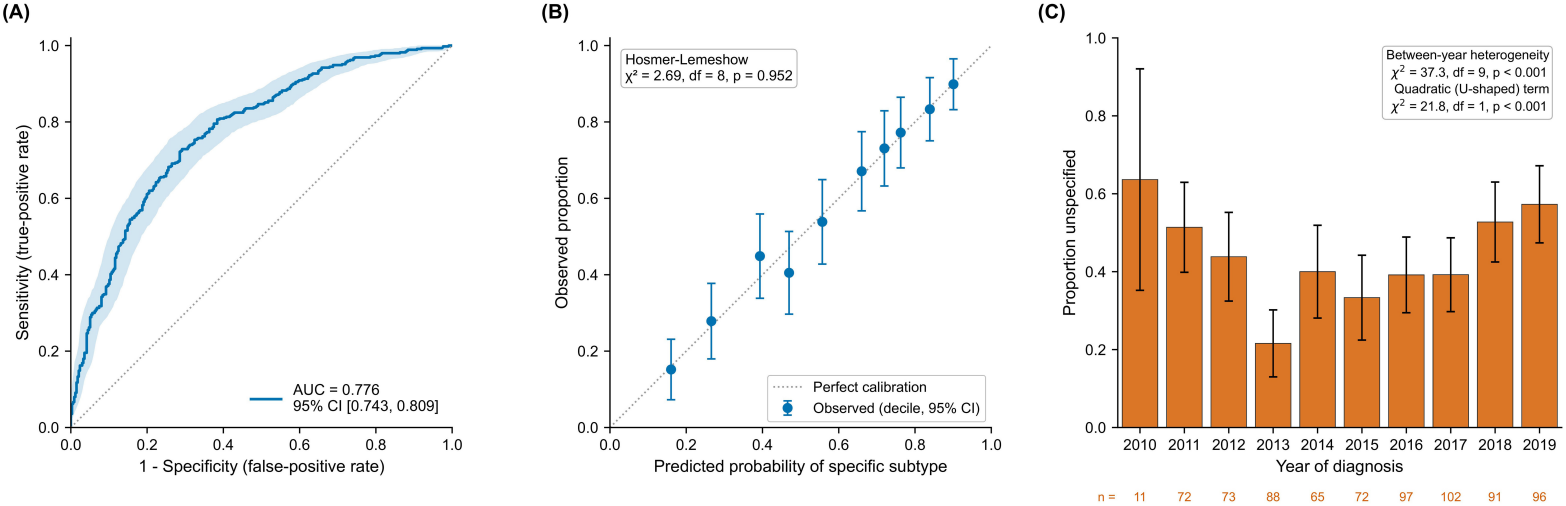
Performance of the primary multivariable logistic model and temporal trend in unspecified diagnoses. **(A)** Receiver operating characteristic (ROC) curve. AUC = 0.776 (95% CI = 0.743, 0.809; percentile bootstrap, 2,000 resamples). The shaded band shows the bootstrap-resampled ROC envelope (1,000 resamples). **(B)** Calibration plot by deciles of predicted probability; error bars are 95% Wald confidence intervals. Hosmer–Lemeshow Chi-square = 2.69; *df* = 8; *p* = 0.952. **(C)** Annual proportion of unspecified diagnoses (95% Wald CI). The sample size per year is shown beneath each bar. This panel is restricted to years of diagnosis from 2010 to 2019 (*n* = 767); 19 patients with recorded diagnosis dates in adjacent years (2008–2009 or 2020–2021) were included in all regression models but are omitted here owing to sparse annual cell counts. The proportion varied significantly across years (likelihood-ratio Chi-square = 37.3; *df* = 9; *p* < 0.001) in a nonmonotonic, U-shaped pattern (quadratic year term Chi-square = 21.8; *df* = 1; *p* < 0.001).

The primary multivariable model achieved an AUC of 0.776 (95% CI = 0.743, 0.809; **Figure 2A**). The bootstrap-resampled ROC envelope was narrow, and the apparent and optimism-corrected AUC values differed by 0.020 (corrected AUC = 0.756; **Supplementary Table 2**), indicating limited overfitting. The L1-penalised model retained 12 of 16 covariates (**Supplementary Table 3**), dropping four whose unpenalised estimates were imprecise or close to the null (consultation-liaison, neurology clinic, psychiatry inpatient, and the psychotic symptom cluster), thereby broadly supporting the dominance of the main factors. Calibration was close to the identity line by decile plot, and the Hosmer–Lemeshow test gave no evidence of poor fit (Chi-square = 2.69; *df* = 8; *p* = 0.952; **Figure 2B**). Variance inflation factors were below 2 for every covariate (**Supplementary Table 4**).

### 3.4 Temporal trend

The proportion of unspecified diagnoses across calendar years is shown in **Figure 2C**. The pattern was nonmonotonic: the annual proportion declined from 0.51 in 2011 to a minimum of 0.22 in 2013 and then rose to 0.57 in 2019, with the earliest year (2010) based on only 11 patients and having a correspondingly wide confidence interval (0.35 to 0.92). The proportion differed significantly across calendar years (likelihood-ratio Chi-square = 37.3; *df* = 9; *p* < 0.001), and a logistic model confirmed significant curvature, with a positive quadratic year term indicating a U-shaped trajectory (Chi-square = 21.8; *df* = 1; *p* < 0.001) that was substantially stronger than the weak linear component (Chi-square = 4.1; *df* = 1; *p* = 0.043). The year-by-brain-scan interaction was nonsignificant (*p* = 0.301; **Supplementary Figure 2**), indicating that the association between imaging and unspecified diagnosis did not vary significantly across the decade. The temporal pattern is therefore better interpreted as showing a decline followed by a rise, which may reflect unmeasured shifts in case mix, referral pathways, or documentation practice rather than a monotonic change.

### 3.5 Subgroup analysis: patients who underwent brain imaging

Among the 484 patients who underwent brain imaging, 139 (28.7%) still received an unspecified diagnosis. The logistic regression restricted to this subgroup used the same covariates as the primary model, with brain imaging held constant at “performed”. The model is reported in **Table 3** and identified the same pattern of associations as the full cohort. Three or more comorbidities (aOR = 0.39; 95% CI = 0.25, 0.62; *p* < 0.001), emergency department entry (aOR = 0.21; 95% CI = 0.10, 0.45; *p* < 0.001), other route entry (aOR = 0.27; 95% CI = 0.14, 0.50; *p* < 0.001), and neurology inpatient entry (aOR = 0.34; 95% CI = 0.15, 0.78; *p* = 0.011) all retained inverse independent associations with subtype determination. Within this subgroup, age was positively associated with subtype determination (aOR = 1.03 per year; 95% CI = 1.01, 1.05; *p* = 0.008), although this subgroup-specific direction should be interpreted cautiously. The reduced model AUC was 0.727 (95% CI = 0.679, 0.777).

**Table 3:** Adjusted logistic regression analysis for factors associated with subtype determination amongst patients who underwent brain imaging (*n* = 484).

Covariate	Adjusted OR	95% CI	*p*-value
Female sex	0.72	[0.47, 1.12]	0.145
Emirati nationality	0.67	[0.43, 1.06]	0.089
Three or more comorbidities	0.39	[0.25, 0.62]	< 0.001
Behavioural symptom cluster	0.6	[0.33, 1.12]	0.109
Psychotic symptom cluster	0.55	[0.23, 1.33]	0.185
Affective symptom cluster	3.53	[0.99, 12.56]	0.052
Vegetative/motor symptom cluster	0.61	[0.31, 1.20]	0.149
Psychiatry inpatient route	0.87	[0.08, 9.16]	0.908
Neurology clinic route	0.7	[0.35, 1.42]	0.326
Neurology inpatient route	0.34	[0.15, 0.78]	0.011
Emergency department route	0.21	[0.10, 0.45]	< 0.001
Consultation-liaison route	1.06	[0.31, 3.64]	0.927
Other route	0.27	[0.14, 0.50]	< 0.001
Age at diagnosis (years)	1.03	[1.01, 1.05]	0.008
Year of diagnosis (centred)	0.95	[0.88, 1.03]	0.248

Of the 484 patients who underwent brain imaging, 139 (28.7%) still received an unspecified label. Reference categories: female sex (male), Emirati (non-Emirati), three or more comorbidities (one to two), symptom cluster (memory-led), location (psychiatry clinic). Brain imaging was held constant (yes) and is therefore not estimated. Expressed as the odds of an unspecified label, the reciprocals are as follows: three or more comorbidities: 2.54 (95% CI = 1.61, 4.03); emergency department: 4.76 (95% CI = 2.23, 10.17); other route: 3.72 (95% CI = 1.99, 6.96); neurology inpatient 2.93 (95% CI = 1.28, 6.69). AUC = 0.727 (95% CI = 0.679, 0.777; percentile bootstrap, 2,000 resamples); Nagelkerke *R*^2^ = 0.191.

### 3.6 Sensitivity and robustness

Five sensitivity scenarios are reported in **Supplementary Table 5**, including one in which the comparator was restricted to the four well-populated subtypes, thereby excluding the sparsely populated categories (frontotemporal, Lewy body, and posterior cortical atrophy dementias). The brain-imaging effect remained large and significant across all scenarios (aOR = 3.82 to 4.04), the comorbidity effect remained consistent (aOR = 0.32 to 0.42), and the emergency department and other location effects retained significance (aOR = 0.27 to 0.30 and 0.28 to 0.32, respectively). The behavioural-symptom effect lost significance only when patients aged over 100 years were excluded (aOR = = 0.65; 95% CI = 0.41, 1.01; *p* = 0.058), suggesting marginal sensitivity to the upper age boundary. Multiple imputation for the 31 cases with missing comorbidity count (*m* = 20) produced pooled estimates essentially indistinguishable from the complete-case primary model (**Supplementary Table 6**).

### 3.7 Clinical-utility analysis

**Figure 3:**
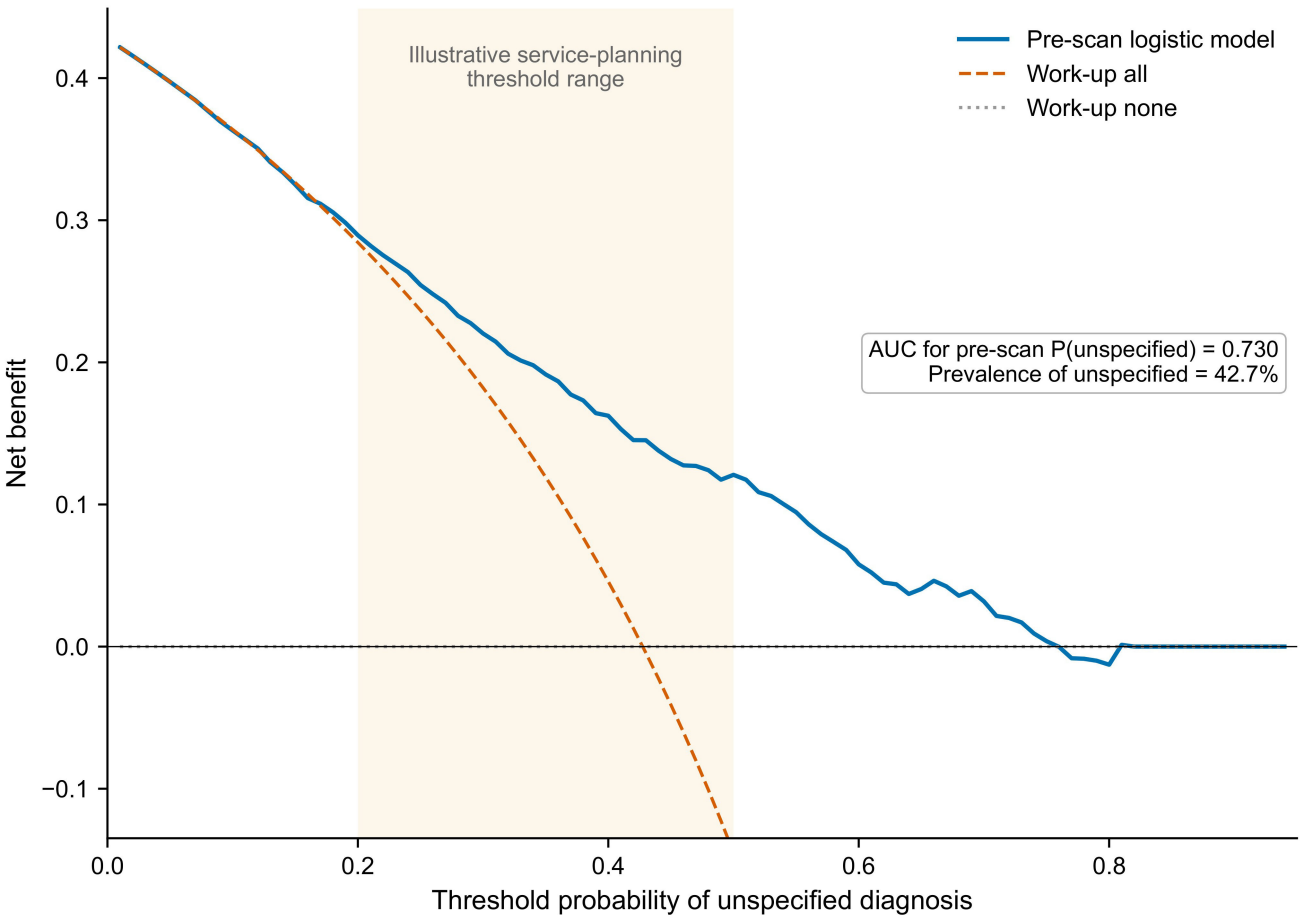
Decision-curve analysis of the prescan logistic model predicting the risk of an unspecified dementia diagnosis. Net benefit is plotted across threshold probabilities of an unspecified diagnosis. The prescan model predicts the probability of an unspecified diagnosis from demographic, symptom, comorbidity, location, and year-of-diagnosis covariates and excludes brain imaging. Reference strategies are work-up all, in which every patient is treated as being at risk of an unspecified diagnosis, and work-up none, in which no patient is treated as being at risk. The shaded vertical band indicates an illustrative service-planning threshold range of 0.20 to 0.50, representing values at which a service might consider prioritising additional subtype-determination work-up, depending on local resources and the perceived cost of leaving a patient with an unspecified diagnosis.

Decision-curve analysis of the prescan logistic model predicting risk of an unspecified dementia diagnosis is presented in **Figure 3**. Across an illustrative service-planning threshold range of 0.20–0.50, the prescan model produced positive net benefit and exceeded both reference strategies, although the margin over work-up all was small at the lower bound (0.289 versus 0.284 at a threshold of 0.20) and widened as the threshold increased. At a threshold of 0.40, the model gave a net benefit of 0.162 versus 0.046 for work-up all and 0 for work-up none, a 3.5-fold improvement over work-up all; net benefit and the corresponding net reduction in unnecessary work-up across the full range of thresholds are reported in **Supplementary Table 9**. The pre-scan model AUC was 0.730.

### 3.8 Exploratory analyses

The symptom-cluster by brain-scan interaction approached but did not reach the conventional threshold of statistical significance (likelihood-ratio Chi-square = 8.27; *df* = 4; *p* = 0.082; **Supplementary Figure 3**). Point estimates suggested that brain imaging was associated with a lower predicted probability of an unspecified diagnosis across symptom clusters; however, confidence intervals were wide, and the apparent between-cluster differences should be interpreted cautiously.

An exploratory product-of-coefficients analysis examined whether the behavioural-versus-memory-led association was partly expressed through brain-scan utilisation (**Supplementary Figure 4**). Within the 616 patients with memory-led or behavioural presentations, the product of the exposure-mediator and mediator-outcome coefficients was − 0.60 on the log-odds scale (bootstrap 95% CI = − 1.32, 0.02). The interval crossed zero, so any indirect pathway through brain imaging is suggested but not confirmed. As effects do not decompose additively on the logistic scale, this is reported descriptively and not as a formal mediation decomposition.

## 4 Discussion

### 4.1 Principal findings

This study treated specific dementia subtype assignment as an indicator of diagnostic completeness with direct relevance to psychiatric care and asked which patient and service-pathway factors were associated with it. In this hospital-based dementia sample, the principal finding was that specific subtype assignment was strongly associated with whether brain imaging had been performed, with imaging being the strongest independent correlate of a specific label. By contrast, three or more comorbidities, behavioural presentation, and entry through the emergency department, other, or neurology inpatient routes were associated with lower odds of subtype determination, whilst age, sex, nationality, year of diagnosis, and the remaining symptom clusters did not retain independent effects (**Tables 1**, **2**).

### 4.2 Imaging access and diagnostic completeness

The imaging association is clinically plausible and consistent with the role of structural neuroimaging in dementia assessment. Imaging can exclude mass lesions, normal-pressure hydrocephalus, and other potentially reversible or alternative causes of cognitive decline, and contributes to subtype differentiation by identifying hippocampal atrophy, vascular burden, strategic infarction, frontotemporal patterns, and posterior cortical involvement. The present finding should therefore be interpreted as evidence that imaging is associated with diagnostic specificity in routine care, rather than proof that imaging alone causes a diagnostic shift.

Imaging in this cohort was not randomly assigned and may have been influenced by clinical severity, referral route, contraindications, patient stability, clinician judgement, documentation practice, and local access constraints (6–9, 25). The central role of structural imaging may also be partly specific to this diagnostic setting, in which molecular and imaging biomarkers were not routinely available. In settings where cerebrospinal fluid or positron-emission-tomography biomarker pathways are established, subtype tends to be recorded more completely, and aetiological classification may depend less on structural imaging; therefore, the determinants of diagnostic completeness may differ in biomarker-rich settings.

Importantly, the absence of imaging did not fully explain unspecified labelling: amongst scanned patients, more than a quarter still received an unspecified diagnosis, and comorbidity burden and entry through the emergency department, other, or neurology inpatient routes remained associated with lower odds of subtype determination (**Table 3**). This subgroup result is central to the interpretation: imaging appears to be important, but not sufficient. Diagnostic specificity may also depend on how imaging is integrated into clinical review, specialist interpretation, follow-up, and documentation.

### 4.3 Clinical complexity, symptom presentation, and care pathways

The multimorbidity association is clinically credible. Patients with three or more comorbidities had markedly lower odds of specific subtype determination in the primary model and in the scanned-subgroup analysis. Multimorbidity is common in dementia and can complicate diagnostic formulation by introducing competing causes of cognitive impairment, overlapping neuropsychiatric or vascular symptoms, polypharmacy, frailty, delirium risk, and competing clinical priorities during time-limited encounters. However, direct evidence specifically linking multimorbidity burden to unspecified dementia labelling remains limited. The most cautious interpretation is therefore that this study identifies multimorbidity as a potential pathway barrier to diagnostic specificity, rather than establishing a causal mechanism (26, 27).

Behavioural symptom presentation was a secondary but noteworthy signal. Behavioural presentations were more common in the unspecified group, and the association with lower subtype assignment persisted after adjustment and multiple imputation. This may reflect the diagnostic ambiguity of behavioural presentations, possible attribution to primary psychiatric disturbance, or differences in work-up pathways. However, the finding was less robust than the imaging, comorbidity, emergency department, and other route associations: it was not significant in the scanned subgroup and became borderline when patients older than 100 years were excluded. It should therefore be framed as hypothesis-generating rather than as a dominant determinant of subtype assignment.

The pathway findings extend the interpretation beyond imaging access alone. Compared with first assessment in the psychiatry clinic, the emergency department route showed the lowest adjusted odds of subtype determination, followed by the other route and the neurology inpatient route. This pattern suggests that non-clinic, acute, heterogeneous, or inpatient entry points may be less conducive to subtype-oriented dementia formulation than planned specialist outpatient assessment. The lower odds amongst patients first assessed on neurology inpatient services are, at first sight, unexpected, given that neurology is itself a specialist setting, but the data suggest a possible explanation: neurology inpatients (*n* = 49) were enriched for cerebrovascular disease (38.8% versus 20.5% at the psychiatry clinic, *n* = 195), consistent with an acute, medically complex admission case-mix, such as stroke, in which the dementia subtype is unlikely to be the focus of the encounter. Consistent with this, only the neurology inpatient coefficient reached significance relative to the psychiatry clinic, whereas the neurology outpatient clinic coefficient did not (*n* = 125; aOR = 0.81; 95% CI = 0.47, 1.41); a direct statistical contrast between the two neurology routes was not tested, and comorbidity burden did not distinguish them.

Imaging was performed in most neurology inpatients (83.7%), indicating that the lower subtype-assignment rate was not explained by the binary imaging indicator alone. The timing, modality, quality, interpretation, and availability of imaging at the point of diagnostic recording were not captured. Definitive subtyping may also have been deferred to later outpatient follow-up or recorded subsequently at the psychiatry reference clinic. The clinical and imaging profile of each entry route is summarised in **Supplementary Table 10**. This interpretation remains hypothesis-generating. Directly comparable evidence on emergency department entry and subtype nonassignment is sparse, but the finding is coherent with the literature showing more frequent nonspecific coding in nonspecialist contexts and incomplete subtype recording in general hospital data. The practical implication is not that acute routes are inappropriate, but rather that patients first recognised through them may require explicit postacute diagnostic review to ensure that the initial broad label does not become the final diagnostic endpoint (10–13).

### 4.4 Implications for psychiatric practice and prescribing safety

The completeness of subtype diagnosis has direct consequences for psychiatric care. Subtype informs whether specific pharmacotherapies are indicated, including cholinesterase inhibitors and memantine and, increasingly, amyloid-targeting disease-modifying therapies for Alzheimer’s disease (28, 29); how vascular risk is managed; and how behavioural and psychological symptoms are interpreted and treated. Behavioural and psychological symptoms affect the majority of people with dementia during the course of their illness (4). It also bears on prescribing safety, because antipsychotic sensitivity in dementia with Lewy bodies means that treating behavioural symptoms without a clear subtype can cause avoidable harm (5). An unspecified label is therefore not a neutral documentation gap but a clinical state in which psychotropic decisions may be made with incomplete information. In the present cohort, first assessment in the psychiatry clinic, the reference pathway, was associated with higher odds of a specific subtype label than the emergency department, other, and neurology inpatient routes, whilst behavioural presentations were associated with lower odds. Read together, these findings suggest that structured specialist psychiatric assessment may support diagnostic completeness and that behavioural presentations, which are common in old age psychiatry, may be at particular risk of remaining nonspecific. Embedding old age psychiatry input and structured behavioural assessment within dementia pathways may therefore improve both diagnostic specificity and the safety of subsequent psychotropic prescribing.

### 4.5 Trends over time

The nonmonotonic temporal pattern requires cautious interpretation. Published trends are mixed: some population-linked datasets show decreasing use of unspecified dementia diagnoses over time, whereas hospitalisation-based analyses suggest that nonspecific dementia codes may dominate inpatient data and that diagnostic coding can drift from specific to nonspecific labels. In the present study, the year-by-brain-scan interaction was nonsignificant, and predicted trajectories by imaging status were approximately parallel, indicating that the imaging-unspecified association did not materially vary across the period. The pattern, a decline to a nadir in 2013 followed by a rise, is therefore better interpreted as potentially reflecting local changes in referral mix, case complexity, hospital workflow, documentation behaviour, or coding practice, rather than a steady trend in either direction (30).

### 4.6 Toward prescan risk stratification

The decision-curve analysis adds a potentially informative but still internally validated dimension. The pre-scan model, which excluded brain-scan status and predicted the risk of an unspecified diagnosis from demographic, symptom, comorbidity, location, and year variables, produced positive net benefit and exceeded both reference strategies across thresholds of 0.20 to 0.50, with the margin over the work-up all strategy narrow at the lower bound and widening as the threshold increased. As the outcome is the risk of remaining with an unspecified dementia diagnosis, the relevant service-level decision is whether to prioritise enhanced subtype-determination work-up, specialist review, imaging completion, or structured follow-up for patients at higher predicted risk. These findings suggest the potential value of an exploratory prescan triage approach for organising dementia pathways, but external validation is required before implementation (20, 31, 32). Future work should test whether structured postimaging or postacute review reduces nonspecific labelling and should examine whether improved diagnostic completeness translates into better treatment selection, counselling, continuity of care, and patient outcomes.

### 4.7 Strengths and limitations

Several strengths increase the value of the study. It focuses on an under-recognised but clinically consequential outcome in dementia research and old age psychiatry: not incidence, mortality, or symptom burden, but whether the care pathway produces a specific subtype assignment, an indicator of diagnostic completeness that directly affects psychiatric management and that has not, to our knowledge, been characterised in any previously published United Arab Emirates or Gulf cohort. The analysis distinguishes the timing of clinical recognition from subtype determination, since duration of undiagnosed symptoms did not differ between groups and was retained only as a descriptive variable. The modelling strategy was clinically interpretable and supported by internal validation, calibration assessment, sensitivity analyses, multiple imputation, and a scanned-subgroup analysis. Robustness analyses were broadly concordant with the primary model: brain scan, comorbidity burden, emergency department route, and other route remained stable across sensitivity scenarios and after imputation.

The study also has important limitations. As a single psychiatry-led hospital site, the findings may not generalise to primary care, private-sector settings, memory clinics with standardised biomarker pathways, neurology-led services, or other hospitals in the United Arab Emirates and the Gulf region. The determinants identified here, imaging access and route of first assessment, are nonetheless structural features shared by many psychiatry-led dementia services in the region that operate without routine biomarker pathways. By contrast, in memory-clinic or neurology-led services with standardised imaging and biomarker access, the differences between entry routes may be attenuated. The cohort was defined by a dementia diagnosis rather than by an age threshold, so a small number of patients were younger than 65 years; this reflects real-world practice, including young-onset presentations seen in psychiatric settings, but means that the sample is not restricted to late-life dementia. The outcome was based on routine diagnostic labelling rather than on uniform expert adjudication; some unspecified diagnoses may represent genuine diagnostic uncertainty, whilst others may reflect incomplete documentation, coding habits, limited follow-up, or unavailable investigation results. Some specific labels are syndromic rather than strictly aetiological: posterior cortical atrophy, for example, is a clinical syndrome that can arise from more than one underlying pathology, so its classification as specific reflects the completeness of the recorded diagnosis rather than confirmed neuropathology, consistent with our outcome definition. Brain imaging was not randomised, and the observed association may be confounded by clinical selection, severity, contraindications, and service access. Imaging modality (computed tomography versus magnetic resonance imaging) was not systematically recorded and so could not be examined, although magnetic resonance imaging may support subtype differentiation more than computed tomography. The Other route category was heterogeneous and should be interpreted as a marker of less clearly specified pathway entry. Recorded age at diagnosis ranged from 23 to 117 years; 21 patients (2.7%) were older than 100, and three were younger than 40. These extreme values could not be verified against source documentation and may reflect data-entry error; excluding patients older than 100 did not materially change the findings, and these records should be interpreted cautiously. Some location strata were sparse, particularly the psychiatry inpatient. The decision-curve findings are internally informative but require external validation. Finally, the study can identify associations and pathway bottlenecks but cannot estimate the causal effect of imaging, route modification, or enhanced follow-up on subtype assignment.

## 5 Conclusion

In this hospital-based dementia cohort, brain imaging was the strongest correlate of specific subtype assignment, but imaging alone did not eliminate nonspecific labelling: clinical complexity and acute or nonspecialist entry routes continued to matter even amongst scanned patients. Diagnostic completeness was therefore more strongly associated with imaging utilisation, clinical complexity and route of entry than with the measured demographic characteristics. As subtype assignment guides psychotropic prescribing and the management of behavioural and psychological symptoms, improving completeness is also a patient-safety priority for old age psychiatry, and these pathway-level findings warrant external validation across other United Arab Emirates and Gulf settings.

## Data Availability

The original contributions presented in the study are included in the article/**Supplementary Material**. Further inquiries can be directed to the corresponding author.
