# Supplementary material for: Why dementia is often not diagnosed by subtype: care pathways and imaging access in the United Arab Emirates
Source: Front Psychiatry. 2026 Aug 5;17:1910567. doi: 10.3389/fpsyt.2026.1910567 (PMC13485901; doi:10.3389/fpsyt.2026.1910567)
Supplement: Supplementary file 1 [file DataSheet1.docx]

**Supplementary Material**

**Supplementary Table S1. Variable coding and recoding scheme.**

| **Variable in** **dataset** | **Recoded as** | **Type** | **Coding rule** | **Reference category** |
| --- | --- | --- | --- | --- |
| Outcome_specific | Dementia subtype diagnosis | Binary | Specific subtype = 1 (Alzheimer's, Vascular, Parkinson's, Lewy body, Frontotemporal, Mixed, Posterior cortical); unspecified = 0. | unspecified |
| Year_dx (continuous), Year_dx_c (centred on median) | Date of dementia diagnosis | Continuous | Calendar year extracted from date. | Median (2015) for centred version |
| Age (continuous), Age_65plus (binary) | Age at diagnosis | Continuous + Binary | Continuous age; binary cut at 65 years for sensitivity. | Age < 65 for the descriptive binary |
| Brain_scan | Brain scan | Binary | Yes = 1, No = 0. | No |
| Female | Sex | Binary | Female = 1, Male = 0. | Male |
| Emirati | Nationality | Binary | Emirati = 1, all other nationalities = 0. | Non-Emirati |
| Comorb_3plus | Number of comorbid conditions | Binary | '3 or more' = 1, '1 or 2' = 0. | 1 or 2 |
| Symptom_cluster | Initial symptom cluster | 5-level categorical | Memory-led (Forgetfulness); Behavioural (Agitation, Irritability, Disinhibition); Psychotic (Delusions, Hallucinations); Affective (Depression, Anxiety, Apathy); Vegetative/motor (Motor disturbance, Appetite, Nighttime behaviours). | Memory-led |
| Location | Location of first assessment | 7-level categorical | Categories: Psychiatry clinic, Psychiatry inpatient, Neurology clinic, Neurology inpatient, ED, CL, Other. 'Other' comprises heterogeneous or incompletely specified locations not among the six named categories, and is treated as a labelled limitation. | Psychiatry clinic |
| DUS_months | Duration of undiagnosed symptoms (from reported symptom onset to dementia diagnosis), months | Continuous (descriptive only) | Reported in Table 1 for descriptive comparison. |  |

**Supplementary Table S2. Bootstrap distribution of adjusted odds ratios and optimism-corrected AUC.**

| **Covariate** | **Apparent aOR** | **Bootstrap mean aOR** | **Bootstrap 95% CI lower** | **Bootstrap 95% CI upper** |
| --- | --- | --- | --- | --- |
| Female sex | 0.846 | 0.84 | 0.601 | 1.199 |
| Emirati nationality | 0.757 | 0.754 | 0.525 | 1.093 |
| Brain scan performed | 4.002 | 4.155 | 2.915 | 5.994 |
| Three or more comorbidities | 0.404 | 0.393 | 0.275 | 0.56 |
| Behavioural symptom cluster | 0.602 | 0.596 | 0.369 | 0.953 |
| Psychotic symptom cluster | 0.889 | 0.905 | 0.439 | 2.054 |
| Affective symptom cluster | 1.603 | 1.689 | 0.802 | 4.157 |
| Vegetative/motor symptom cluster | 0.764 | 0.762 | 0.433 | 1.362 |
| Psychiatry inpatient route | 1.649 | Not estimable | Not estimable | Not estimable |
| Neurology clinic route | 0.811 | 0.809 | 0.448 | 1.458 |
| Neurology inpatient route | 0.444 | 0.438 | 0.207 | 0.915 |
| Emergency Department route | 0.286 | 0.276 | 0.153 | 0.505 |
| Consultation-Liaison route | 1.131 | 1.223 | 0.444 | 4.02 |
| Other route | 0.307 | 0.3 | 0.183 | 0.484 |
| Age at diagnosis, per year | 1.005 | 1.006 | 0.989 | 1.022 |
| Year of diagnosis, centred on 2015 | 0.993 | 0.993 | 0.932 | 1.055 |

**Note.** Non-parametric bootstrap with 2,000 attempted bootstrap resamples; 1,869 successful. Apparent AUC 0.776; mean optimism 0.020; optimism-corrected AUC 0.756. Estimates for sparse strata are unstable: for the Psychiatry inpatient route the bootstrap distribution did not converge to a usable range, and the resampled mean and confidence interval are therefore reported as not estimable. The apparent odds ratio for this stratum is retained for transparency and should not be interpreted substantively.

**Supplementary Table S3. LASSO-penalised logistic regression versus the primary unpenalised model.**

| **Variable** | **LASSO coefficient (standardised)** | **Retained** | **Primary adjusted OR** | **Primary adjusted p value** |
| --- | --- | --- | --- | --- |
| Age at diagnosis, per year | 0.021 | 1 | 1.005 | 0.4963 |
| Brain scan performed | 1.336 | 1 | 4.002 | <0.001 |
| Three or more comorbidities | -0.867 | 1 | 0.404 | <0.001 |
| Emirati nationality | -0.206 | 1 | 0.757 | 0.1135 |
| Female sex | -0.093 | 1 | 0.846 | 0.3154 |
| Consultation-Liaison route | 0.0 | 0 | 1.131 | 0.8074 |
| Emergency Department route | -1.003 | 1 | 0.286 | <0.001 |
| Neurology clinic route | 0.0 | 0 | 0.811 | 0.4591 |
| Neurology inpatient route | -0.492 | 1 | 0.444 | 0.0242 |
| Other route | -1.005 | 1 | 0.307 | <0.001 |
| Psychiatry inpatient route | 0.0 | 0 | 1.649 | 0.6715 |
| Affective symptom cluster | 0.288 | 1 | 1.603 | 0.2659 |
| Behavioural symptom cluster | -0.383 | 1 | 0.602 | 0.0247 |
| Psychotic symptom cluster | 0.0 | 0 | 0.889 | 0.7514 |
| Vegetative/motor symptom cluster | -0.148 | 1 | 0.764 | 0.336 |
| Year of diagnosis, centred on 2015 | -0.015 | 1 | 0.993 | 0.8295 |

**Note.** L1-penalised logistic regression with a 10-fold cross-validated penalty parameter. Continuous covariates were standardised before penalisation, so LASSO coefficients are on a standardised scale; the primary adjusted odds ratios are on the original scale (per year for age and for year of diagnosis). Variables with non-zero LASSO coefficients are flagged as Retained = 1. Concordance with the primary unpenalised model is reported alongside.

**Supplementary Table S4. Primary multivariable logistic model diagnostics and variance inflation factors.**

| **A. Primary multivariable logistic model diagnostics** | |
| --- | --- |
| **Diagnostic** | **Value** |
| Number of observations (analytic n) | 786 |
| Log-likelihood (full model) | -439.62 |
| Log-likelihood (null) | -536.52 |
| Cox-Snell R² | 0.219 |
| Nagelkerke R² | 0.293 |
| AUC (apparent) | 0.776 |
| AUC 95% CI (bootstrap, B = 2000) | [0.743, 0.809] |
| Hosmer-Lemeshow χ² | 2.69 |
| Hosmer-Lemeshow degrees of freedom | 8 |
| Hosmer-Lemeshow p value | 0.952 |
| **B. Variance inflation factors** | |
| **Variable** | **VIF** |
| Intercept | 66.67 |
| Age at diagnosis | 1.11 |
| Female sex | 1.03 |
| Emirati nationality | 1.06 |
| Brain scan performed | 1.13 |
| Three or more comorbidities | 1.08 |
| Year of diagnosis, centred on 2015 | 1.09 |
| Behavioural symptom cluster | 1.12 |
| Psychotic symptom cluster | 1.06 |
| Affective symptom cluster | 1.12 |
| Vegetative/motor symptom cluster | 1.08 |
| Psychiatry inpatient route | 1.04 |
| Neurology clinic route | 1.52 |
| Neurology inpatient route | 1.21 |
| Emergency Department route | 1.36 |
| Consultation-Liaison route | 1.15 |
| Other route | 1.8 |

**Note.** AUC, area under the receiver operating characteristic curve; CI, confidence interval; VIF, variance inflation factor. Discrimination was assessed using the AUC, with the 95% CI obtained from a non-parametric bootstrap with 2,000 resamples. Calibration was assessed using the Hosmer-Lemeshow goodness-of-fit test. VIFs are reported for each design-matrix column; the intercept is shown by convention but is not interpreted. All non-intercept VIFs were below 2, well within the conventional target threshold of 5.

**Supplementary Table S5. Sensitivity analyses for the primary logistic model.**

| **Scenario** | **n** | **AUC** | **Covariate** | **aOR** | **95% CI** | **p** |
| --- | --- | --- | --- | --- | --- | --- |
| 1. Primary: unspecified vs all specific subtypes | 786 | 0.776 | Brain scan | 4.0 | [2.84, 5.63] | <0.001 |
| 1. Primary: unspecified vs all specific subtypes | 786 | 0.776 | ≥ 3 comorbidities | 0.4 | [0.29, 0.57] | <0.001 |
| 1. Primary: unspecified vs all specific subtypes | 786 | 0.776 | Behavioural cluster | 0.6 | [0.39, 0.94] | 0.025 |
| 1. Primary: unspecified vs all specific subtypes | 786 | 0.776 | Location: ED | 0.29 | [0.16, 0.51] | <0.001 |
| 1. Primary: unspecified vs all specific subtypes | 786 | 0.776 | Location: Other | 0.31 | [0.20, 0.48] | <0.001 |
| 1. Primary: unspecified vs all specific subtypes | 786 | 0.776 | Age (per year) | 1.01 | [0.99, 1.02] | 0.496 |
| 1. Primary: unspecified vs all specific subtypes | 786 | 0.776 | Female sex | 0.85 | [0.61, 1.17] | 0.315 |
| 1. Primary: unspecified vs all specific subtypes | 786 | 0.776 | Emirati nationality | 0.76 | [0.54, 1.07] | 0.114 |
| 2. Unspecified vs Alzheimer's disease only | 653 | 0.785 | Brain scan | 3.82 | [2.61, 5.60] | <0.001 |
| 2. Unspecified vs Alzheimer's disease only | 653 | 0.785 | ≥ 3 comorbidities | 0.32 | [0.22, 0.46] | <0.001 |
| 2. Unspecified vs Alzheimer's disease only | 653 | 0.785 | Behavioural cluster | 0.51 | [0.31, 0.84] | 0.008 |
| 2. Unspecified vs Alzheimer's disease only | 653 | 0.785 | Location: ED | 0.27 | [0.15, 0.51] | <0.001 |
| 2. Unspecified vs Alzheimer's disease only | 653 | 0.785 | Location: Other | 0.28 | [0.17, 0.45] | <0.001 |
| 2. Unspecified vs Alzheimer's disease only | 653 | 0.785 | Age (per year) | 1.01 | [1.00, 1.03] | 0.108 |
| 2. Unspecified vs Alzheimer's disease only | 653 | 0.785 | Female sex | 0.9 | [0.63, 1.29] | 0.579 |
| 2. Unspecified vs Alzheimer's disease only | 653 | 0.785 | Emirati nationality | 0.81 | [0.56, 1.18] | 0.275 |
| 3. Unspecified vs four well-populated subtypes | 775 | 0.774 | Brain scan | 4.03 | [2.86, 5.68] | <0.001 |
| 3. Unspecified vs four well-populated subtypes | 775 | 0.774 | ≥ 3 comorbidities | 0.41 | [0.29, 0.58] | <0.001 |
| 3. Unspecified vs four well-populated subtypes | 775 | 0.774 | Behavioural cluster | 0.6 | [0.38, 0.93] | 0.023 |
| 3. Unspecified vs four well-populated subtypes | 775 | 0.774 | Location: ED | 0.3 | [0.17, 0.54] | <0.001 |
| 3. Unspecified vs four well-populated subtypes | 775 | 0.774 | Location: Other | 0.32 | [0.20, 0.50] | <0.001 |
| 3. Unspecified vs four well-populated subtypes | 775 | 0.774 | Age (per year) | 1.01 | [0.99, 1.02] | 0.471 |
| 3. Unspecified vs four well-populated subtypes | 775 | 0.774 | Female sex | 0.85 | [0.62, 1.18] | 0.344 |
| 3. Unspecified vs four well-populated subtypes | 775 | 0.774 | Emirati nationality | 0.75 | [0.53, 1.06] | 0.099 |
| 4. Strict handling: records with a missing outcome label excluded from the complete-case sample (n = 782) | 782 | 0.775 | Brain scan | 4.04 | [2.87, 5.70] | <0.001 |
| 4. Strict handling: records with a missing outcome label excluded from the complete-case sample (n = 782) | 782 | 0.775 | ≥ 3 comorbidities | 0.41 | [0.29, 0.58] | <0.001 |
| 4. Strict handling: records with a missing outcome label excluded from the complete-case sample (n = 782) | 782 | 0.775 | Behavioural cluster | 0.62 | [0.39, 0.96] | 0.033 |
| 4. Strict handling: records with a missing outcome label excluded from the complete-case sample (n = 782) | 782 | 0.775 | Location: ED | 0.28 | [0.16, 0.51] | <0.001 |
| 4. Strict handling: records with a missing outcome label excluded from the complete-case sample (n = 782) | 782 | 0.775 | Location: Other | 0.31 | [0.20, 0.49] | <0.001 |
| 4. Strict handling: records with a missing outcome label excluded from the complete-case sample (n = 782) | 782 | 0.775 | Age (per year) | 1.01 | [0.99, 1.02] | 0.479 |
| 4. Strict handling: records with a missing outcome label excluded from the complete-case sample (n = 782) | 782 | 0.775 | Female sex | 0.84 | [0.60, 1.16] | 0.289 |
| 4. Strict handling: records with a missing outcome label excluded from the complete-case sample (n = 782) | 782 | 0.775 | Emirati nationality | 0.76 | [0.54, 1.07] | 0.119 |
| 5. Excluding patients older than 100 years | 765 | 0.776 | Brain scan | 4.0 | [2.83, 5.66] | <0.001 |
| 5. Excluding patients older than 100 years | 765 | 0.776 | ≥ 3 comorbidities | 0.42 | [0.30, 0.60] | <0.001 |
| 5. Excluding patients older than 100 years | 765 | 0.776 | Behavioural cluster | 0.65 | [0.41, 1.01] | 0.058 |
| 5. Excluding patients older than 100 years | 765 | 0.776 | Location: ED | 0.29 | [0.16, 0.53] | <0.001 |
| 5. Excluding patients older than 100 years | 765 | 0.776 | Location: Other | 0.32 | [0.21, 0.51] | <0.001 |
| 5. Excluding patients older than 100 years | 765 | 0.776 | Age (per year) | 1.0 | [0.98, 1.01] | 0.785 |
| 5. Excluding patients older than 100 years | 765 | 0.776 | Female sex | 0.87 | [0.63, 1.22] | 0.423 |
| 5. Excluding patients older than 100 years | 765 | 0.776 | Emirati nationality | 0.77 | [0.54, 1.09] | 0.138 |

**Note.** Five sensitivity scenarios. Scenario 1 is the primary specification (reference). Scenarios 2 and 3 restrict the comparator to Alzheimer's disease only or to the four well-populated specific subtypes. Scenario 4 applies strict missing-data handling. Scenario 5 excludes patients aged over 100 years.

**Supplementary Table S6. Pooled estimates from multiple imputation (m = 20) for missing comorbidity count.**

| **Covariate** | **Pooled aOR** | **95% CI** | **p** |
| --- | --- | --- | --- |
| Female sex | 0.87 | [0.631, 1.199] | 0.395 |
| Emirati nationality | 0.761 | [0.542, 1.069] | 0.115 |
| Brain scan performed | 4.171 | [2.983, 5.832] | <0.001 |
| Three or more comorbidities | 0.414 | [0.295, 0.581] | <0.001 |
| Behavioural symptom cluster | 0.597 | [0.386, 0.922] | 0.02 |
| Psychotic symptom cluster | 0.921 | [0.450, 1.886] | 0.823 |
| Affective symptom cluster | 1.592 | [0.715, 3.541] | 0.255 |
| Vegetative/motor symptom cluster | 0.803 | [0.468, 1.380] | 0.427 |
| Psychiatry inpatient route of first assessment | 1.114 | [0.189, 6.561] | 0.905 |
| Neurology clinic route of first assessment | 0.826 | [0.480, 1.421] | 0.489 |
| Neurology inpatient route of first assessment | 0.437 | [0.219, 0.873] | 0.019 |
| Emergency Department route of first assessment | 0.28 | [0.159, 0.491] | <0.001 |
| Consultation-Liaison route of first assessment | 1.195 | [0.447, 3.195] | 0.723 |
| Other route of first assessment | 0.323 | [0.210, 0.498] | <0.001 |
| Age at diagnosis, per year | 1.007 | [0.992, 1.022] | 0.359 |
| Year of diagnosis, centred on 2015 | 0.996 | [0.939, 1.056] | 0.885 |

**Note.** Multiple imputation was performed for missing comorbidity count among records otherwise eligible for the imputation analysis. Although 32 records in the source cohort had missing comorbidity count, one of these also had missingness in another model covariate and was therefore not included in the imputation working sample; 31 records were imputed. Iterative chained imputation with a Bayesian ridge estimator and posterior sampling was used (m = 20). Pooled estimates were obtained using Rubin’s rules. Conclusions were concordant with the complete-case primary model in **Table 2**.

**Supplementary Table S7.** Pre-scan logistic model: intercept and coefficient estimates on the log-odds scale.

| **Covariate** | **Coefficient (log-odds)** | **Standard error** | **Adjusted OR** | **95% CI** |
| --- | --- | --- | --- | --- |
| Intercept | −2.0106 | 0.6214 | 0.1339 | [0.0396, 0.4526] |
| Age at diagnosis, per year | 0.0006 | 0.0075 | 1.0006 | [0.9859, 1.0154] |
| Three or more comorbidities | 0.7954 | 0.1633 | 2.2153 | [1.6086, 3.0509] |
| Behavioural cluster | 0.5872 | 0.2133 | 1.7989 | [1.1843, 2.7323] |
| Psychotic cluster | 0.0254 | 0.3551 | 1.0257 | [0.5114, 2.0572] |
| Affective cluster | −0.6001 | 0.4118 | 0.5488 | [0.2448, 1.2301] |
| Vegetative/motor cluster | 0.1576 | 0.2661 | 1.1707 | [0.6949, 1.9722] |
| Psychiatry inpatient | −0.6230 | 1.1513 | 0.5363 | [0.0562, 5.1220] |
| Neurology clinic | 0.2631 | 0.2717 | 1.3010 | [0.7639, 2.2157] |
| Neurology inpatient | 0.6397 | 0.3522 | 1.8958 | [0.9506, 3.7810] |
| Emergency Department | 1.3770 | 0.2817 | 3.9629 | [2.2817, 6.8829] |
| Consultation-Liaison | −0.1768 | 0.4843 | 0.8379 | [0.3243, 2.1650] |
| Other route | 1.4496 | 0.2184 | 4.2615 | [2.7777, 6.5377] |
| Year of diagnosis, centred | −0.0068 | 0.0296 | 0.9932 | [0.9372, 1.0525] |

**Note**. Pre-scan logistic regression model fitted with all covariates except brain-scan, used to enable decision-curve analysis (**Figure 3**) and operationalise the decision of whether to prioritise additional subtype-determination work-up, defined as imaging completion, memory clinic review, post-acute structured diagnostic review, coding review, or multidisciplinary team review. Reference categories follow **Table 2** of the main manuscript: male sex, non-Emirati nationality, 1 or 2 comorbidities, memory-led symptom cluster, and Psychiatry clinic location of first assessment. Note that the pre-scan model is fitted on the reverse-coded outcome (unspecified = 1, Specific subtype = 0) to support the decision-curve analysis in **Figure 3**, so adjusted odds ratios above 1 indicate higher odds of an unspecified diagnosis; the direction of effects is therefore inverted relative to **Table 2**. The apparent pre-scan model AUC was 0.730 in the complete-case analytic sample. Coefficient values, standard errors, adjusted odds ratios, and 95% confidence intervals are reported from the pre-scan logistic model fit on the complete-case analytic sample (n = 786). The fitted equation is: logit(P(unspecified = 1)) = −2.011 + 0.001·Age + 0.207·Female + 0.350·Emirati + 0.795·(≥3 comorbidities) + 0.587·Behavioural + 0.025·Psychotic − 0.600·Affective + 0.158·Vegetative/motor − 0.623·Psychiatry inpatient + 0.263·Neurology clinic + 0.640·Neurology inpatient + 1.377·Emergency Department − 0.177·Consultation-Liaison + 1.450·Other − 0.007·(Year − 2015). McFadden's pseudo-R² was 0.119; the model converged with no covariates dropped.

**Supplementary Table S8. Prevalence of recorded comorbid conditions by subtype-determination status (analytic n = 786).**

| **Comorbid condition** | **Overall, n (%)** | **Unspecified (n = 336), n (%)** | **Specific (n = 450), n (%)** | **p value** |
| --- | --- | --- | --- | --- |
| Diabetes mellitus | 465 (59.2) | 198 (58.9) | 267 (59.3) | 0.967 |
| Hypertension | 530 (67.4) | 250 (74.4) | 280 (62.2) | <0.001 |
| Cerebrovascular disease | 220 (28.0) | 90 (26.8) | 130 (28.9) | 0.569 |
| Falls or mobility problems | 276 (35.1) | 136 (40.5) | 140 (31.1) | 0.008 |
| Chronic kidney disease | 145 (18.4) | 63 (18.8) | 82 (18.2) | 0.924 |
| Parkinsonism | 48 (6.1) | 10 (3.0) | 38 (8.4) | 0.003 |
| Mental health condition | 47 (6.0) | 25 (7.4) | 22 (4.9) | 0.180 |
| Chronic liver disease | 4 (0.5) | 3 (0.9) | 1 (0.2) | 0.423 |
| Other or rare | 118 (15.0) | 74 (22.0) | 44 (9.8) | <0.001 |

**Note.** Conditions were recorded across up to three comorbidity fields per patient; the highest category was therefore top-coded as three or more conditions. Comparison by Pearson's chi-square. Parkinsonism was more frequent among subtyped patients, consistent with its role in defining Parkinson's disease dementia. Comorbidity burden was higher in the unspecified group (mean 2.53 versus 2.24 recorded conditions; Mann-Whitney p < 0.001).

**Supplementary Table S9. Net benefit of the pre-scan model across threshold probabilities of an unspecified diagnosis.**

| **Threshold probability** | **Harm-to-benefit ratio** | **Net benefit: model** | **Net benefit: work-up all** | **Net benefit: work-up none** | **Net reduction in unnecessary work-ups per 100** |
| --- | --- | --- | --- | --- | --- |
| 0.10 | 1:9.0 | 0.363 | 0.364 | 0.000 | -0.6 |
| 0.20 | 1:4.0 | 0.289 | 0.284 | 0.000 | 2.0 |
| 0.30 | 1:2.3 | 0.220 | 0.182 | 0.000 | 8.9 |
| 0.40 | 1:1.5 | 0.162 | 0.046 | 0.000 | 17.5 |
| 0.50 | 1:1.0 | 0.121 | -0.145 | 0.000 | 26.6 |
| 0.60 | 1:0.7 | 0.058 | -0.431 | 0.000 | 32.6 |

**Note.** The threshold probability encodes the harm-to-benefit trade-off; no monetary inputs are required. Net reduction in unnecessary work-ups is the number avoided per 100 patients relative to a work-up-all strategy at the same net benefit. The model's advantage over work-up-all emerges from a threshold of approximately 0.20 upward.

**Supplementary Table S10. Clinical and imaging profile by location of first assessment (analytic n = 786).**

| **Location of first assessment** | **n** | **Specific subtype, n (%)** | **Cerebrovascular disease, %** | **3 or more comorbidities, %** | **Brain scan performed, %** | **Mean age, y** |
| --- | --- | --- | --- | --- | --- | --- |
| Psychiatry clinic | 195 | 148 (75.9) | 20.5 | 41.5 | 73.8 | 76.7 |
| Psychiatry inpatient | 6 | 5 (83.3) | 16.7 | 33.3 | 83.3 | 70.3 |
| Neurology clinic | 125 | 86 (68.8) | 28.8 | 64.8 | 72.0 | 75.9 |
| Neurology inpatient | 49 | 30 (61.2) | 38.8 | 59.2 | 83.7 | 76.9 |
| Emergency Department | 93 | 43 (46.2) | 29.0 | 44.1 | 55.9 | 79.1 |
| Consultation-Liaison | 30 | 23 (76.7) | 36.7 | 50.0 | 80.0 | 73.1 |
| Other | 288 | 115 (39.9) | 29.9 | 59.4 | 44.4 | 81.1 |

**Note.** Percentages are within each entry route. This table supports the interpretation of the neurology-inpatient finding: that route carried the highest cerebrovascular burden and a high comorbidity load, indicating an acute, medically complex case-mix, despite the highest imaging rate.

**Supplementary Figure S1. Univariate odds ratios for factors associated with specific subtype determination.**

**Note.** Forest plot of unadjusted odds ratios with 95% confidence intervals on a logarithmic x-axis. The vertical reference line at OR 1 indicates no association. Estimates whose confidence interval excludes 1 are highlighted in colour; estimates spanning 1 are shown in grey.

**Supplementary Figure S2. Year of diagnosis by brain-scan interaction effect on the predicted probability of an unspecified diagnosis.**

**Note.** Predicted probability of an unspecified diagnosis from a logistic model including a year by brain-scan interaction term, holding all other covariates at their observed sample distribution. The two trajectories run approximately parallel, indicating that the observed association between brain imaging and unspecified diagnosis did not vary significantly across the study period (interaction p = 0.301). The annual analysis covers years of diagnosis 2010-2019 (n = 767); 19 patients with recorded diagnosis dates in adjacent years were included in all regression models but omitted from the annual panels for sparse cell counts.

**Supplementary Figure S3. Initial symptom cluster by brain-scan interaction effect on the predicted probability of an unspecified diagnosis.**

**Note**. Predicted probability of an unspecified diagnosis by symptom cluster and brain-scan status. Error bars are bootstrap 95% confidence intervals (B = 500). Imaging was associated with lower predicted probability of unspecified diagnosis across symptom clusters. The interaction did not reach statistical significance, and apparent between-cluster differences should be interpreted cautiously because several confidence intervals were wide. Likelihood-ratio chi-square 8.27, df = 4, p = 0.082.

**Supplementary Figure S4. Exploratory product-of-coefficients decomposition of the behavioural-versus-memory-led association with subtype determination via brain-scan utilisation.**

**Note.** Exploratory product-of-coefficients analysis restricted to patients presenting with memory-led or behavioural symptoms (n = 616). Path a (exposure to mediator) and path b (mediator to outcome) are reported as log-odds-ratios, with bootstrap resampling based on B = 2,000 replicates. Because the outcome model is logistic, the total, direct, and indirect quantities are not additively collapsible; the product a × b is reported as an exploratory summary only, and no total-effect decomposition is claimed. Confidence intervals for the product a × b are bootstrap percentile intervals (B = 2,000 resamples).
